# The Pathogenesis of Tabes Dorsalis

**Published:** 1911-01

**Authors:** T. A. Williams

**Affiliations:** Washington, D. C.; Neurologist to Epiphany Dispensary; Member Corresp. Loc. de Neurol. de Paris; oc. de Psychol. de Paris; Memb. Assoc. Soc. Clin. de Med. Ment., Etc.


### THE PATHOGENESIS OF TABES DORSALIS.

By Dr. T. A. Williams, Washington, D. C.

*Neurologist to Epiphany Dispensary; Member Corresp. Loc. de Neurol. de Paris; oc. de Psychol. de Paris; Memb. Assoc. Soc. Clin. de Med. Ment., Etc.*

The pathogenesis of Tables Dorsalis has excited a great deal of discussion ever since Duchenne (5), first differentiated the locomotor ataxia he so well describes. None of the more recent reviews of the subject have formulated very definite conclusions nor acceded categorically to a single theory. None of the framers of these have quite succeeded in accounting for all the clinical facts already ascertained.

Having had exceptional opportunities during the last two years (1908) of examining the preparations of many observers and of carefully going over a very large number of cases of Tabes fruste, I wish in this presentation to indicate as briefly as possible the reasons for the conclusions reach that in the main the true explanation reached is that of Nageotte (2). An attempt is made also to account for the discrepancies between the findings of others and those of his remarkable series of studies upon the radicular nerve.

Let us avoid all arbitrary divisions, types, stages of disease, sketching as far as possible the processes which occur and the evolution of the symptoms, postulating in the first place with the great majority of neurologists that the tabetic process differs not in kind but only in locality from that of general paralysis; and in the second place to reject the explanation familiar in the text books that tabes is a primary distrophy either of the posterior column or of the sensory protoneurone as a whole or an atrophy thereof directly caused either by alcohol, prolonged strain, syphilis or sexual excesses.

To make this clear a few anatomical and physiological details must be re-called.

It will be recalled that in or near the row of foramina, between the laminae of the vertebrae, are situated the ganglia upon the posterior roots of the spinal cord and that the lower ganglia are a considerable distance inside the spinal canal. The nutritional function of the ganglion cells is too well known to require emphasis. But it is, perhaps, insufficiently borne in mind that the regenerative function of these cells is efficacious only when the fibre concerned possesses a neurilemmia, and that within the spinal cord the axons of the root-nerves lose this sheath, retaining only the white substance of Schwann.

Between the lower ganglia and the segment of the spinal cord, corresponding with each, there intervenes several inches which the radicular nerve traverses in contact with the leptomeninges, whose arrangement is shown in Fig. 1. (Am. Jour. Med. Sci.) This shows clearly the canal formed by the pachymeningeal reflection as the nerve pierces the theca vertebralis to become peripheral. In this canal, it is probable that communication takes place between the cerebro spinal fluid filling it, and the lymph in the sheat of the peripheral nerve. (Orr and Rows) (1).

Figures 2, 3, 4, 5 and 6 show the lesions described by Nageotte (69), in this locality in cases of tables and tabo-paralysis. Examination shows this lesion in all stages from simple round cell infiltration, through granuloma, towards necrosis with cavity formation. They are the fruit of complete serial sections of several radicular nerves in each of eleven cases of this disease.

Now even the chronicity of the infection and the comparatively indolent reaction of the granulomatous process are in this situation sufficient to seriously affect the nerve fibres contiguous to the inflammatory area; for in the circumscribed canal of unyielding dura-mater, the pressure of toxic effects of the exudate impinges upon the nerve fibres surrounding. These are either destroyed, or at least sufficiently injured to prevent the passage of the impulses from the ganglion cells which subserves the trophicity of the distal portion of the fibre, which accordingly degenerates. Their lack of neurilemma prevents their regeneration, even after the granuloma has undergone absorption; and the post-mortem appearance of tabes dorsalis is thus constituted. Regeneration, however, does occur in the posterior root itself just as far as Obersteiner's ring; and it is to this cause that we must attribute the many attempts to refer the tabetic process to a primary dystrophy. For the earlier pathologists had not conceived that what appeared a healthy structure, was the real source of the lesion they found in the posterior column of the spinal cord.

Nageotte's (2), opinion that the process is toxic does not accord with the findings of similar posterior column degenerations with evidence of pressure upon the roots, in cases of cerebral tumor (Lejonne 3), in which the manometric pressure of the spinal fluid is increased; and it would seem that the mechanical factor of the granulomatous infiltration was enough to account for th e tabes, although the experiments of Orr and Rows (4), show that causes of purely toxic in kind may produce degenerative changes.

The preponderance of the process upon the fibers of deep sensibility is connected by the name given to the disease by Duchenne (5), of Boulogne, loco-motor ataxia. Its occurrence may be due to as yet unascertained peculiarities in the course of these fibers, which we know to accompany the motor nerves. A search among carefully described cases of tabes, however, shows that other anomalies of sensation are very much commoner than the classics allow; it is probable, therefore, that it is merely the inconvenience entailed by the impairment of this function which is the cause of the stress laid upon its involvement when the pathology of tabes is considered.

Figures 3 and 4 show how desperate is the involvement of the fasciculi composing the radicular nerve, Whether this is due to an elective affinity of a toxine for a particularly sensory system, or whether it is due to an accidental incidence depending on the local differences of arrangement, cannot be affirmed with certainty. Clinical evidence seems to indicate that some of the fibres subserving the sensation of cold are spared relatively long; but no accurate data yet exists to indicate whether these fibres belong to the protopathic or epicritic systems demarkated by Head (6.)

The fibres subserving the sense of attitude and those of deep pain are considered to be particularly early affected; and Marinesco (7), has shown that the former is generally associated with the appreciation of vibration, which is much impaired in the course of tabes.

Now Head (8), has shown that these are two functions of deep fibres accompanying motor nerves. Spacing sense, thought by him to be in part a function of the same system, has not yet been sufficiently investigated in tabetics, but the implication of the two foregoing shows that the lesion must be one of the peripheral sensory neurone, for these two modalities of sensation become disassociated in the spinal cord, and would require two separate lesions for their implication, sense of position and movement passing homolaterally in the posterior column, while deep pain joins the superficial pain to pass hetero-laterally in a portion of Gowers tract (Page May (83.)

Touch and deep pressure impulses are, on the other hand, amalgamated into a single path as soon as they reach their relay neurone in the spinal cord. They mount cephalad in a hetero-lateral tract in the neighborhood of the ventral horn. Thus, if a lesion situated in the cord prevents the passage of impulses of deep pressure, it necessarily prevents the passage of tactile, cutaneous impulses also, whether these are epicritic or protopathic, these distinctions being solely peripheral as regards their topographic separation. Head and Thompson (9.)

The disparate character of the sensory troubles is shown in "Tabes Superienre" and by such cases as radicular asterognosis as were described by Dejerine (10), and Noica and Pop Averamescu of Bucarest, where the palmer side and corresponding fingers could not detect the bodies palpated.

As other examples of such irregular incidence, the cases cited by Dupre (12) and Camus (13), Dufour may be cited. A most demonstrative case was that of Babinski and Nagotte (47), where the patellarreflex had persisted until death. Post-mortem it was found that the middle root zones were much less affected than the posterior root zones, while the reflex collaterals and the ventral fasciculus of the posterior horn were relatively conserved. This greater implication of the long fibers is according to Nageotte rathe the exception. Raymond's (15), case, too, is striking in this regard, as all deep reflexes were retained.

Although hypotonia usually accompanies the lost tendon reflexes, yet even these are sometimes disassociated, as in this case. It would appear, too, that the hypertonic muscles of the tabetic may become even spastic, either by involvement of the pyramidal tract, as in so-called Tabes Combinee, Noica (16), or by the development of paralysis agitans, as in the case of Bychowski (17). Reflexes, too, may persist for some time, although there is grave disorganization of afferent fibres from muscle, tendon and joint as evidenced by great muscular inco-ordination and lost sense of attitudes.

It is a pity that in the 2 cases of Long (81), the condition of the vestibular nerve was not described, for the preservation of the patellar reflexes was there combined with great ataxia; and yet at post-mortem, very little change was found in the lower spinal cord or roots.

The beautiful description by Gowers (16), of the varying character of the lightning pains well shows the irregularity with which the fibers are attacked in the posterior roots, though his interpretation is inconsistent with the pathology for which this paper contents:

"The superficial pains seem to be on the surface or just beneath the surface. They are usually felt at one spot, often only a few inches in extent, sometimes larger, extending down part of a limb, on the ears, on the face and especially about the mouth.

"The pain is usually extremely brief, a stab or flash of pain, gone as soon as felt, but recurring.

"These superficial pains have on remarkable effect; they make the skin tender. After they have occurred a few times, sometimes almost as soon as they begin, the skin in that area becomes so tender that if touched the patient feels as if the place was an open sore. If the finger is drawn gently over it, or the bedclothes are moved upon it, the pain produced is often hardly bearable. A remarkable fact is that this occurs where sensibility to pain is lost."

This description accords with that of the destruction of spicritic and irritation of the protopathic sensation ("the prick of a pin may occasion no pain whatever.") (6) (Head).

Of the deep pains, the radicular character is as manifest, e. g., pain after pain occurs at the same part, separated by a short interval; it is sometimes described as "like the pain of cramp when there is no muscular contraction." Of the girdle sensation, Gowers says that when superficial, it is often accompanied by a hyperaesthesia, but when deep is without corresponding changes in the skin. Gowers also points out their occasional unilateral character.

The paraesthesia are an endless list, and the false interpretations are very probably due to the different degree of involvement of deep and epicritic fibers. His case of tabetic neuralgia without loss of the knee jerks is a striking confirmation of this point of view, "for they were entirely superficial; he could not bear the prick of a pin, but the sensation of touch was perfect." What is this but a protopathic hyperaesthesia? There were five similar cases, all thus characteristic of root pains. Now reference to Head's hyperaesthesia cases and those described by Lebar (20), will show that hyperesthesia of this nature is not necessarily due, as Gowers thinks, to irritation in the periphery, but in the nerve trunk. The nutritional changes also confirm this point of view. In one case of cutaneous erethema , with numerous small white spots, accompanied the pain; in one, the localized pain of the scalp was followed after a week by bending and breaking of the hairs about an eighth of an inch from their emergence. These phenomena conform to the trophic ones which Head considers as dependent upon protopathic fibers.

The radicular distribution of the trophic changes is not consistent with their peripheral origin. The resemblance, too, of the arthopathy and osteopathy to those of syringo-myelia is striking, but in the latter case the lesion affects the vaso-centers in the inter-medio-lateral column, and possibly certain other root fibers also, whereas in the tabetic process it is the conducting fibers alone which are involved. The effect, however, does not differ. That these dystrophies are dependent upon the interruption of afferent impulses is, however, shown by the disappearance of the perforating ulcer when rest to the limb is enforced for a few weeks. It is only then that the ulcer can be adequately protected from the unfelt traumata which interferes with its healing. Such an exposed surface would normally be so painful that the patient would be compelled to rest; but the painless mal-perforante offers no inducement to this.

One of the hitherto unrecognized paresthesia is pruritis. Its characters as pointed out by Lamy and Millian (22), strikingly exemplify the irregular incidence of the tabetic process, for though generally bilateral, it may be confined to one side, and it usually coincides with segmentala area presenting other symptoms such as hypo-aesthesia, or visceral crises. It naturally occurs only during the earlier stages of the disease.

The researches of Sherrington (23), and others, have shown that the motor nerves of the anatomist are also accompanied by sensory fibres. These fibres proceed from end organs within the substance of the muscles, known as the muscle spindles. These spindles are sensitive to the deformation they undergo upon contraction of the muscles. It is possible that they are more exposed to noxious meningeal influences than other sensory fibres, which proceed straight into the posterior root. This, perhaps, explains the frequency of their involvement in the meningeai inflammation which leads to Tabes Dorsalis, though unpublisherd reseaches of Ransome kindly communicated to me by Dr. Donaldson, of the Wistar Institute, show that in rats at least these fibers are well mixed with those of cutaneous sensation by the time they reach the spinal ganglion.

When the clinician investigates the loss of deep pain by pressing the ulnar nerve behind the elbow, pinching the tendon Achilles or faradizing the sural muscles, he is ascertaining the diminution of a function conveyed by these same nerves. Each of these symptoms is very common (90%) (Dejerine) (24), quite early in the disease.

As a matter of fact, the other fibres are not spared, and if the disease is not arrested or cured or ended by death, all forms of sensibility are destroyed. The disease, however, progresses very slowly in most cases, although some rapid cases have been mistaken for acute myelitis, just as fulminating phthisis has for Croupous pneumonia. As in tuberculosis, the individual lesions tend toward arrest, while the process tends to progress, the rate varying with the individual and the circumstances.

This tendency to local arrest explains the infrequency of serious myopathies, for Nageotte (2), has shown that the anterior roots often degenerate.

The possibility of its degeneration has not been generally accepted. But these and other cases as, well as the regeneration of the posterior roots, definitely shown by Bickles' (25), experiments should compel the most recalcitrant to accept the evidence of Nageotte's preparations as regards the anterior roots.

Muscular atrophy is, however, much commoner in Tabes than earlier writers allowed. Witness the cases of Dejerine (72), Souques (71), Marie (72), Oddo (74), Camp (75), Raymond (15), and Nageotte's (2), case of Aran-Duchenne Palsy in a syphilitic, ataxic in the evenings, where the patellar reflexes had disappeared, who showed lost photomotor reflexes (ididoplegia), and lymphocytosis, and whose symptoms began with ulnar neuralgia, is undoubtedly a tabetic, though the muscular atrophy may, of course, be of independent causation.

The recollection of the pathological principle upon which this depends would have saved many from their undue scepticism in this regard. It is sufficiently well known that nerves possessing a neurilemma will regenerate when no longer shut off from their trophic centre.

Now, the trophic centre of the anterior root is the pyramidal cells in the anterior horn. Upon the absorption of the meningeal exudation which has interrupted that influence, they push out new fibres, which Nageotte has figured, and these, finding no interruption wend their way once more to the muscular fibres which they innervate.

In the posterior root, on the other hand, the portion of the fibre distal from its trophic center,—the spinal ganglion, loses its neurilemma on entering the central nervous system. Having suffered degeneration through the meningeal exudation upon the posterior root that is between itself and its trophic centre, its want of neurilemma deprives it of power to regenerate, even when the trophic centre retains access, for the regenerating fibres, as Bickles' (25), experiments show, stop short at the point of reflection of the pia mater at Obersteiner's ring (figure). Hence, from the effects of this lesion no recovery is possible, but permanent disability through the muscular atrophy due to anterior root lesions occurs only when the inflammatory exudation has eventuated in cicatrization. Still later researches of Marinesco

(80), and Nageotte (2), show that in many cases the regenerating fibers fail to reach the spinal cord, some of them not even emerging from the ganglion.

These conclusion have been reached, thanks in great part to the examination of the "masses en croissance" first described by Cajal (82).

Although I am one of the last to minimize the importance of the psychic factor in the temporary abasia of the tabetic, yet I believe that the future will show that these are sometimes, in part at least, due to the evanescent peripheral neurone palsies induced in the manner described. Faure (27), has already shown that ataxia of the trunk muscles is the real cause of some cases of tabetic abasia which was supposed to be psychic.

David Ferrier (28), in the Lumleian lectures has summarized the objections to these interpretations of the pathology of tabes dorsalis, by stating that the meningitis postulated is not a constant phenomenon, being more frequently absent than present, and when existing, being more of a secondary thickening than an inflammatory process. These objections are completely refuted by the cytological data of lumbar puncture. That lmyhocytosis signifies meningeal inflammation no one disputes.

The frequent post-mortem findings of thickenings rather than inflammations is explained by the law of fibrosis of individual lesions in the history of granulomata, the process itself continuing in other parts.

Ferrier further objects that in tabes well-marked intra-medullary degeneration is often accompanied by neuritis or atrophy of posterior roots. The experiments of Bickles effectively answer this objection, as previously indicated. Ferrier declares unintelligible what he terms the practical escape of the anterior roots, and thinks it contradicts the usual law of the greater involvement of the motor fibres in neuritis of mixed nerves.

This so-called law is an imagination of clinicians, as no one who has studied alcoholic neuritis can doubt, for in this affection of mixed nerves the motor fibres sometimes escape entirely. The fact that the motor neurone is involved in plumbism often to the exclusion of the sensory is due to its greater susceptibility to that particular toxin. Pharmacological studies show the existence of these selective powers. Ferrier, too, declares that it is extremely rare for menegitis to produce intra-spinal degeneration by implication of the spinal roots. This statement is contradicted by the examination of cases of Potts disease, Alquier (28), spinal tumors, etc. Another argument he invokes is the segmental character of tabetic degeneration, but as segmental is a term in this connection convertible with radicular, the logic of this objection is not evident.

Another objection—its symmetrical character does not accord with the facts, for Babinski (29), and Dejerine (24), have shown that in the early stages a relative asymmetry is an important character in the tabetic process.

Another objection is the occurrence of degeneration elsewhere, as in cranial nerves, ciliary ganglion and sympathetic system. The cranial nerve affections are, of course, due to a similar basal mningitis (Williams, Am. Jour. Med., Mar., 1910, while it is not established that implication of the ciliary ganglion is an essential part of the tabetic process.

The pathology of the sympathetic involvement will be considered further on in this paper.

The fact that Thomas 30), in each of six cases found decided changes in the spinal ganglion cells, in no way invalidates the pathogenesis of Nageotte, for "reaction a distance," is a well-ascertained certainty. Besides, some of the cases of Redlich (31), Mari and Leri (32), show distinct, direct involvement of the spinal ganglia by an inflammatory process which has evidently extended from the meninges. This extension is not at all surprising when one remembers that even the purely mechanical lesions of the root, described by Lejonne (3), in cases of cerebral tumor sometimes reach the ganglia.

When the ganglion is directly affected by such an interstitial inflammation, it is not difficult to understand that the function of the cells becomes impaired, or even that their death may ensue. This will be manifested by a dendritic degeneration, which may well begin in the most distant part and hence give rise to the symptoms and post-mortem appearance of the peripheral neuritis in tabes as described by Dejerine (24), and others.

*To Be Continued.*

